# Pre- and peri-natal hurricane exposure alters DNA methylation patterns in children

**DOI:** 10.1038/s41598-023-30645-5

**Published:** 2023-03-08

**Authors:** Erin Kello, Alexandre R. Vieira, Sona Rivas-Tumanyan, Maribel Campos-Rivera, Karen G. Martinez-Gonzalez, Carmen J. Buxó, Evangelia Morou-Bermúdez

**Affiliations:** 1 University of Pittsburgh, Pittsburgh, USA; 2 University of Puerto Rico Medical Sciences Campus, San Juan, Puerto Rico

**Keywords:** Epigenetics, Genetics, Climate sciences, Medical research, Risk factors

## Abstract

Hurricane Maria was the worst recorded natural disaster to affect Puerto Rico. Increased stress in pregnant women during and in the aftermath of the hurricane may have induced epigenetic changes in their infants, which could affect gene expression. Stage of gestation at the time of the event was associated with significant differences in DNA methylation in the infants, especially those who were at around 20–25 weeks of gestation when the hurricane struck. Significant differences in DNA methylation were also associated with maternal mental status assessed after the hurricane, and with property damage. Hurricane Maria could have long lasting consequences to children who were exposed to this disaster during pregnancy.

## Introduction

Puerto Rico (PR) is an archipelago in the Eastern Caribbean with a population of approximately 3.3 million^1^. It has been an un-incorporated territory of the United States since 1898 characterized by high poverty rate (43%) and high income inequality^2^. The island is burdened by significant public health problems, including one of the highest prevalence rates of premature births in the United States (11.8%, 2020) and worldwide^3,4^, and a high incidence of HIV infections. The Zika epidemic also affected the island^5^.

On September 20 of 2017, PR was devastated by Hurricane Maria, a category 4 storm that affected the entire island. Two weeks earlier, Hurricane Irma, a category 5 storm had passed very close to the island causing significant damage on the infrastructure. The storms completely wiped-out the island’s electric power infrastructure, water supplies, communications, and transportation systems (including all ports and airports), leaving many residents isolated for several weeks. By November 2017, two months after the storm, only 50% of the electric power generation had been restored, and 10% of the citizens were still without water service; by February 2018, only 70% of the electric power generation capacity had been restored^5^. In October 2017 it was estimated that there were 51 deaths directly caused by the hurricane, and an additional 900 potentially hurricane-related deaths. Subsequent studies estimated that the “excess all-cause deaths” in Puerto Rico during the three months following Hurricane Maria could amount to more than 4000^6^.

Although it is far too soon to estimate the public health consequences of Hurricane Maria in Puerto Rico, pregnant women were an especially vulnerable population during this unprecedented disaster because of the distress from the fearful experience itself, the disruption of prenatal care, nutritional alterations due to lack of electricity and fresh food supplies, and possible exposures to infectious and toxic materials. During the storm the majority of the 69 major hospitals on the island were left without electricity or fuel for their generators, and only three hospitals were functional 3–4 days after the storm^2^. Although obstetric services had to be available 24/7, not all women had access to them, and the conditions were far from adequate. The University Hospital was fully operational by day 8, and by day 9 it was estimated that the number of deliveries was 33% higher compared to the same month of the previous year^2^. The threat to life and multiple causes of a state of loss associated with this hurricane were a source of increased PNMS that can lead to significant maternal mental health problems like depression and post-traumatic stress disorder (PTSD)^2,6,7^. Dietary changes are also a common consequence in these types of emergencies due to the lack of access to fresh food supplies^8^. The Island’s prevailing high importation rate added to the direct impact of the hurricane on the ports of entry further aggravated the loss of resources available to address basic needs.

Prenatal maternal stress due to natural disasters has been shown to impact practically all spheres of child development, including birth outcomes, and cognitive, motor, physical, socio-emotional, and behavioral development^9,10^. Previous experience from natural disasters like Hurricane Andrew (1992), Hurricane Katrina (2005), the Iowa Floods (2008), the Queensland Floods (2011) and the Ice Storm of Québec (1998) have shown that these types of prenatal stressors can lead to adverse pregnancy outcomes, such as prematurity, reduced birth weight and head circumference and other neonatal complications, especially in highly exposed women^9,11–14^. The long-term health consequences for the children who were prenatally exposed to these events are far more important, and they include effects on early infant temperament and motor development^15^, early cognitive development^16^, increased risk for immunologic conditions such asthma^17^ autism traits^18,19^ and obesity^11^.

Prenatal maternal stress may affect the fetus via the release of maternal stress hormones however, many of the long-term effects of prenatal maternal stress on offspring health can also be attributed to epigenetic changes to the fetal genome^20,21^. Children and adolescents prenatally exposed to the 1998 Québec Ice Storm showed significant differences in methylation levels of thousands of CpG sites, based on their mother’s objective prenatal maternal stress during the storm, and/or her cognitive appraisal of the impact of the storm in her life. The differentially methylated CpGs were affiliated with 1564 different genes and 408 biological pathways, primarily related to immune function^22^. These epigenetic changes were associated with significant health problems later in the life of these children, including obesity and diabetes^23,24^, and, more recently, brain development^25^.

The objective of this study is to evaluate DNA methylation profiles in children who were exposed to Hurricane Maria in Puerto Rico during pregnancy in relation to hurricane-related maternal mental health exposures. Assessments were performed within two years of the exposure to the hurricane using validated measures for objective and subjective maternal stressors. Our hypothesis was that children who were prenatally exposed to maternal psychosocial and environmental stressors associated with Hurricane Maria in Puerto Rico would have an altered DNA methylation profile. We report that the timing of the traumatic maternal event affects methylation patterns differently as expressed in early childhood. Specific measures of maternal stress are also associated with differing methylation patterns. The results of this study provide information for clinical providers working with women who experience a traumatic event during pregnancy. Our findings further inform the current understanding of the temporal dimension of DNA methylation during development by demonstrating differences in methylation levels based on the period of gestation at the time of the major traumatic event.

## Results

A total of 47 significant differentially methylated single probes (DMPs) were associated with all hurricane-related variables tested (Supplemental Table 1).

**Figure 1:**
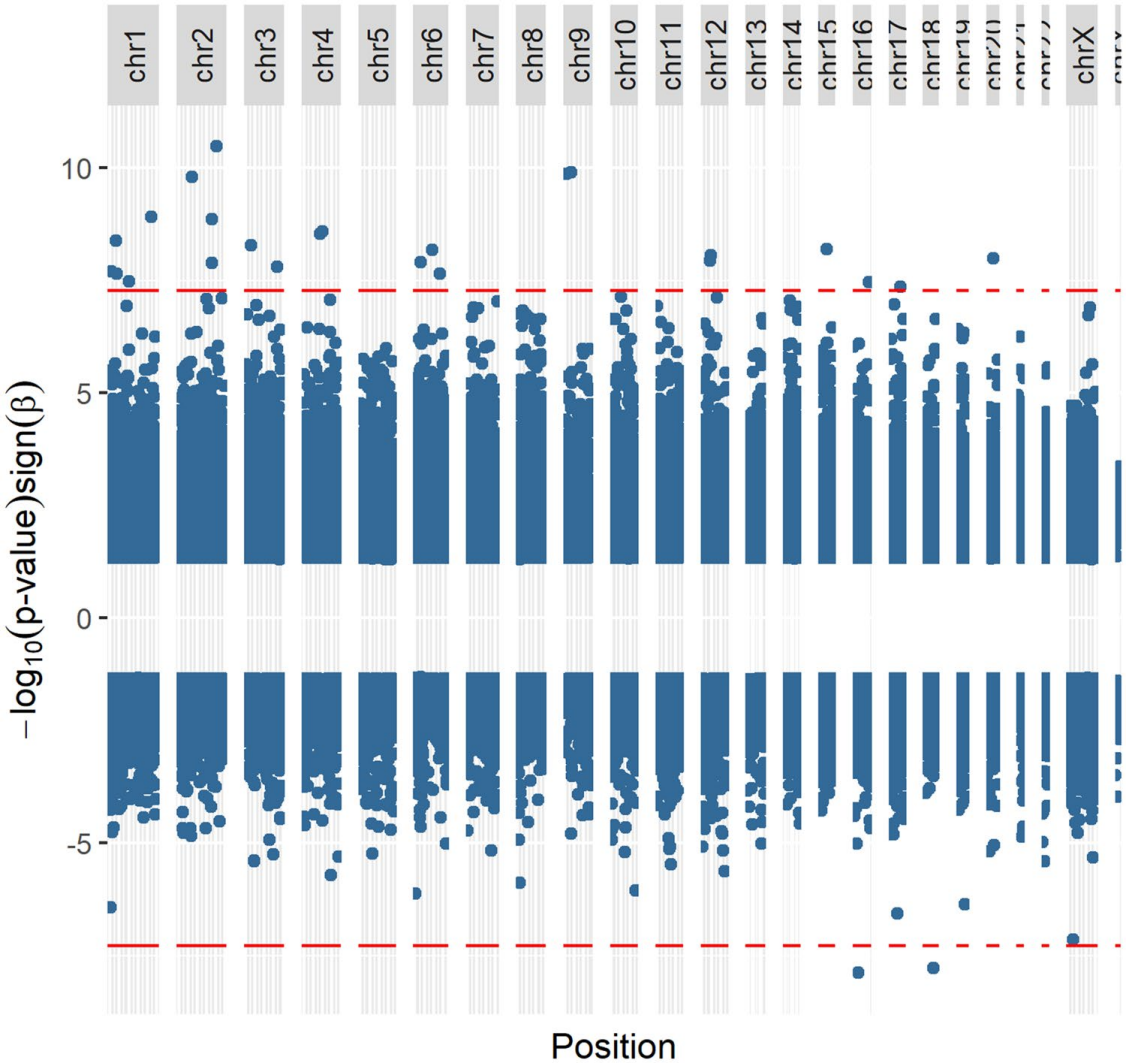
Manhattan plot of significant CpG sites associated with the stage of gestation [weeks] at the time of hurricane impact. Red lines represent FDR significance threshold.

We identified 30 significant DMPs associated with the gestation stage in weeks at the time of the hurricane (Fig. 1). Almost all DMPs significantly associated with the timing of the exposure are hypermethylated. The most significant hypermethylated probes cluster on chromosomes 1–4.

**Figure 2:**
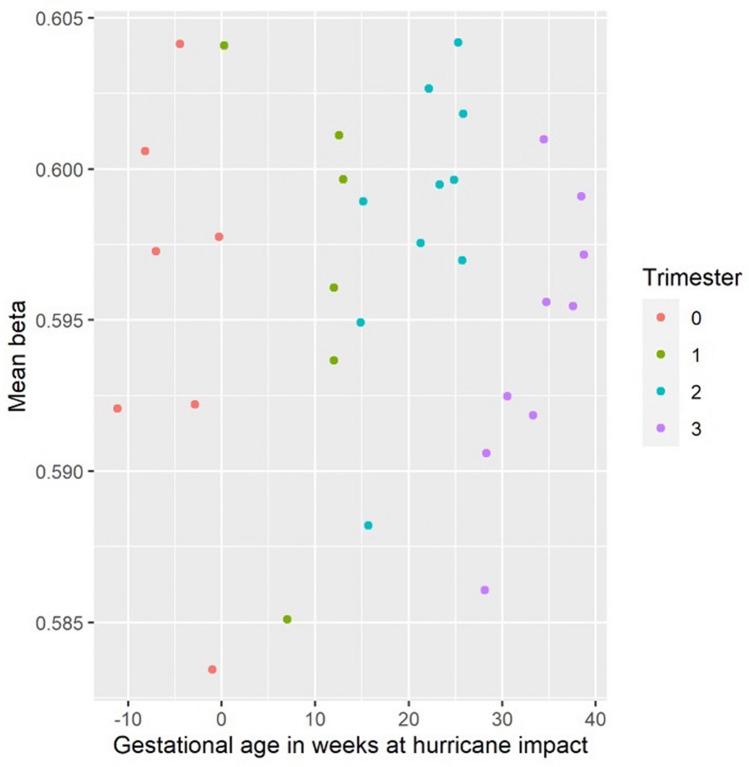
Mean methylation levels by gestational age at impact. Each dot corresponds to a different child. Colors indicate trimester. (0) indicates children conceived within three months after the hurricane.

Mean methylation levels were calculated for each subject and used to explore the direction of methylation changes dependent on the time of maternal exposure to the hurricane. Maternal exposure between 20- and 25-weeks’ gestation yields higher mean methylation levels (Fig. 2). Average methylation values for women that were exposed in the first part of the third trimester (~ 30 weeks) are lower compared to other exposure times (Fig. 2).

One significant true differentially methylated region (DMR) with multiple probes was associated with the timing of the hurricane. This DMR contains four probes and is in the promoter region of *LLRC39* gene. The FDR adjusted p-value for this region is 0.000355. The probes were in the 5′UTR and the TSS200 (within 200 base pairs of the transcription start site) region of the gene. The probes are in open chromatin. A heatmap of this region shows that higher methylation values are found in the children of women who were in the 2nd half (< 20 weeks) of their pregnancy during the hurricane impact (Supplementary Fig. S1). The *LLRC39* gene produces a protein that is a component of the sarcomeric M-band, which plays a role in myocyte response to biomechanical stress^26^. Downregulation of *LRRC39* in spontaneously beating engineered heart tissue results in a lower generation of force. An in-vivo zebrafish knockdown model of *LRRC39* resulted in severely impaired cardiac function and cardiomyopathy^26^.

Of the 10 most significant DMPs associated with the timing of exposure, the most biologically relevant site is the probe located near the *SPR* (Sepiapterin Reductase) gene. This DMP is located within a CpG island and within 200 bps of the transcription start site in an open chromatin region. The enzyme sepiapterin reductase is involved in the production of the tetrahydrobiopterin (BH4), which converts amino acid precursors into the neurotransmitters serotonin and dopamine^27^. BH4 is also a necessary cofactor for nitric oxide (NO) synthesis via nitric oxide synthase (NOS). Mutations in this gene have been linked to hypertension and brachycardia, likely because of an imbalance between sympathetic and parasympathetic input and impaired NO production in endothelial cells^28^. SPR variants have also been linked to a higher susceptibility to bipolar disorder^29^ and an increased risk of schizophrenia in females of Han Chinese descent^30^. Other functions represented by genes associated with significant DMPs include basic cellular processes, apoptosis, and cell proliferation, and the epigenetic process of histone demethylation.

A total of five DMPs were associated with the constructed variable “prenatal maternal stress” (PNMS) (Supplementary Fig. S2). The high PNMS group included children of mothers with symptoms of moderate to severe depression and post-traumatic stress disorder (PTSD), while the low PNMS mothers had no symptoms of depression or PTSD (Supplement: Study Sample). One of these DMPs is associated with the gene *CRIP2*; the same DMP is also significantly associated with the maternal PHQ-9 score. Another DMP significant in the PNMS group, associated with a region on chromosome 10, was also significant in the PTSD group. CRP2, the protein produced by the *CRIP2* gene is associated with smooth muscle differentiation. CRP2 forms a complex with serum response factor and GATA proteins which converts fibroblasts to smooth muscle cells^31^.

**Figure 3:**
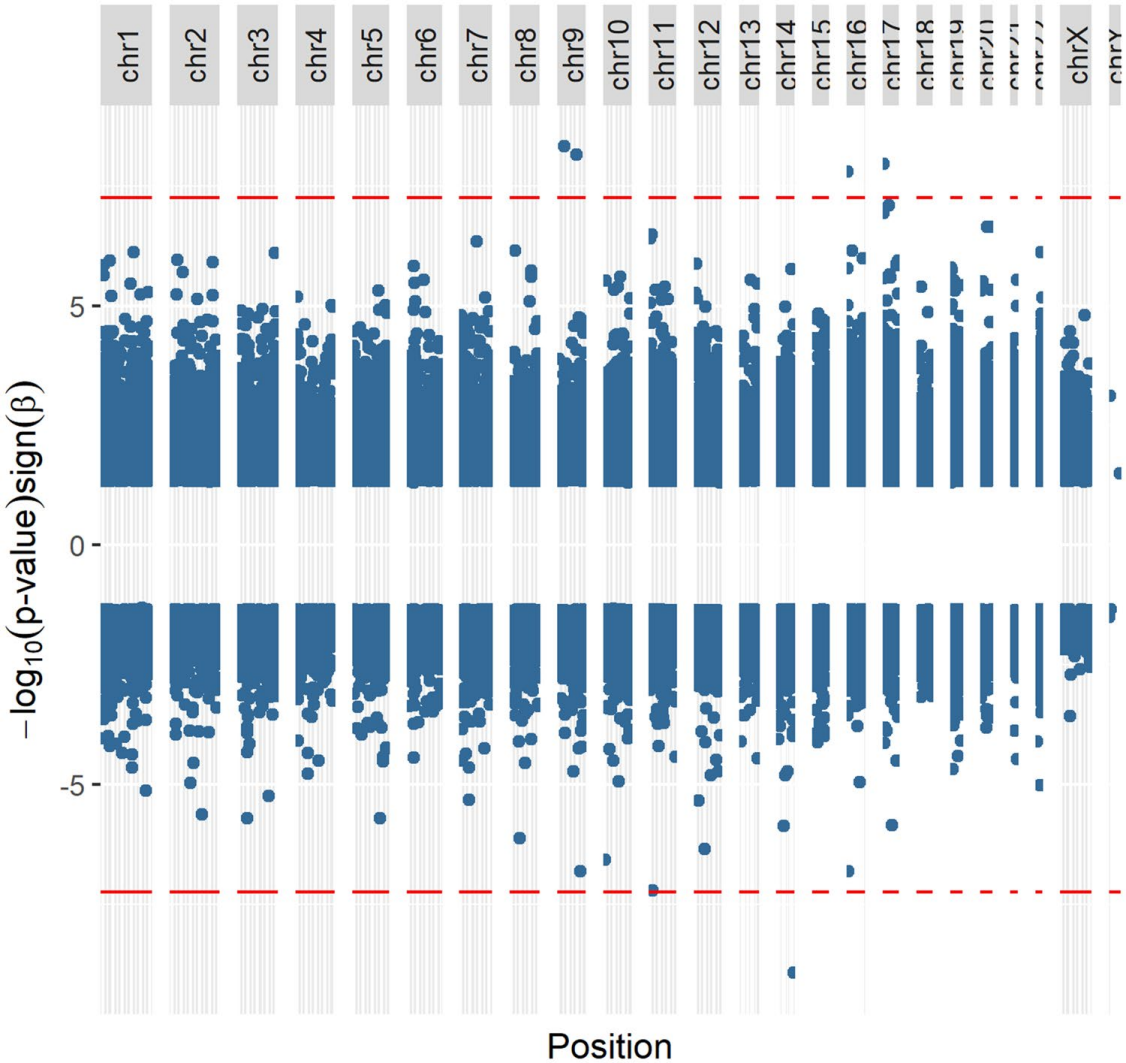
Manhattan plot of significant CpG sites associated with PHQ-9 score, which measures symptoms of depression in the mothers. Red lines represent FDR significance threshold.

Five significant DMPs were associated with the categorical PHQ-9 score, which measures levels of depression symptoms in the mothers (categories: none, mild, moderate, moderately severe, and severe) were identified by the dmrff algorithm (Fig. 3). The significant DMPs map to genes involved in cell proliferation; protein folding, trafficking, prevention of aggregation, and proteolytic degradation; methionine metabolism/ histone methylation, and maintenance of intracellular calcium homeostasis. None of the significant DMPs for the PHQ-9 score are in a CpG-dense region or upstream of the transcription start site. Four of the five significant DMPs are hypermethylated and may represent a pattern of hypermethylation associated with this score.

**Figure 4:**
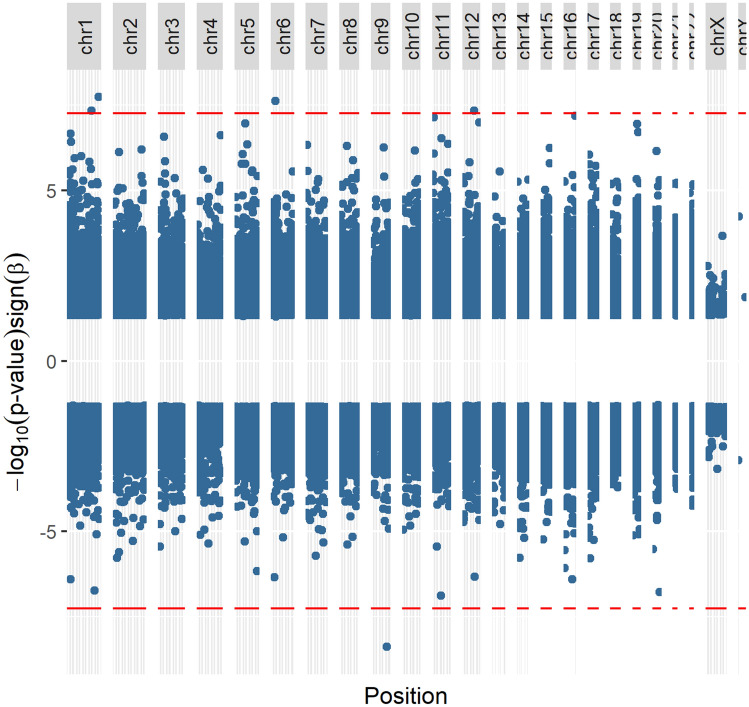
Manhattan plot of significant CpG sites associated with PSS-10 score, which measures symptoms of perceived stress in the mothers. Red lines represent FDR significance threshold.

Five significant DMPs were associated with the categorical PSS-10 score which measures symptoms of perceived maternal stress (categories: High, Moderate, Low) (Fig. 4). None of the significant DMPs are in a CpG dense region or upstream of the transcription start site. The significant DMPs map to genes involved in carbohydrate metabolism, actin cytoskeleton and cell polarity organization, and neuronal migration. Four of the five significant probes are hypermethylated and may represent a pattern of hypermethylation associated with this score.

Three significant DMPs were associated with the binary variable of presence/absence of PTSD symptoms (Supplementary Fig. S3). Of the three significant DMPs, two mapped to genes that are involved in RNA processing. Both DMPs were hypermethylated. One probe, associated with the *HNRNPF* gene is in a CpG island and in the TSS200 or 5′ UTR region depending on alternate splicing variants. The *HNRNPF* gene is part of the heterogeneous nuclear ribonucleoproteins [hnRNPs] family of genes that regulate mRNA processing and transport. The hnRNP family is also involved in regulating telomerase activity and telomere length^32^. Individuals with psychiatric disorders have significantly shorter telomeres^33^. Send et al.^34^ found higher levels of perceived maternal stress during pregnancy associated with shorter telomeres in newborns. Aberrant methylation of HNRNPF associated with maternal PTSD might represent an epigenetic pathway by which psychological maternal trauma is associated with shorter telomeres in offspring.

One significant DMP was associated with the categorical “property damage” score (Supplementary Fig. S4). It is located on chromosome 9, a CpG shore region, with an adjusted p-value of 0.013865217. This DMP is hypomethylated. The DMP is in the body of the *JAK2* gene. The JAK2 protein is important for controlling the production of blood cells from hematopoietic stem cells and gain-of-function mutations have been linked to polycythemia vera and idiopathic erythrocytosis^35^.

Changes in global methylation at repetitive genomic elements have been linked with genomic instability and disease processes^36^. To explore if global methylation changes were associated with exposure to the hurricane, a linear model was applied to a subset of 17,730 probes associated with long interspersed nucleotide element-1 (LINE-1) elements. The PNMS variable (yes/no) was used to assess if there were methylation differences between the groups. No significant differences were found.

## Discussion

The Developmental Origins of Health and Disease theory (DOHaD) postulates that exposure to prenatal stressors during critical periods of early development could lead to permanent alterations of the developing tissues. These alterations could lead to a higher risk of developing serious pathological conditions later in life, such as obesity, cardiometabolic disease, immunological conditions, and mental health problems^20^. Although the biological mechanisms that mediate these effects are not completely understood, recent studies indicate that epigenetic mechanisms are an important “interface” through which the body interprets and responds to stressful experiences early in prenatal and perinatal life by modulating DNA function and gene expression^21^.

Founded on the principals of the DOHaD this study examined the impact of prenatal stress due to Hurricane Maria on the methylation profiles of children participating in the HELiOS cohort (Hurricane Exposures and Long-term Infant Outcomes Study), which includes 187 children exposed to hurricane Maria in Puerto Rico during pregnancy, and their mothers. The epigenetic analysis only included children who were evaluated within 2 years of the disaster, which is much earlier compared to previous studies on other natural disasters^22,37^. The timely evaluation of the mother–child dyads is a strength of this study because it allowed us to identify mothers who experienced higher levels of PNMS due to the disaster and to compare them against those with no apparent symptoms of mental distress. The assessments were performed using validated instruments, providing an additional strength to the study.

The timing of maternal exposure to the hurricane during pregnancy was the factor with the strongest impact on the methylation patterns in children. In the absence of a non-exposed control, this finding provides a strong argument that the observed effects were indeed related to the disaster. Property damage, another objective hurricane-related exposure, was associated with one significant DMP. Subjective measures of PNMS, such as maternal depression, stress, and PTSD symptoms were also associated with differences in methylation patterns in children in our study, but the impact was smaller. Previous studies in children prenatally exposed to the Quebec ice-storm in 1998 have also reported that the objective hardship experienced by the mothers during the natural disaster had a stronger association with the DNA methylation patterns of affected children, compared to more subjective maternal distress levels^22^. These observations reflect the difficulty in accurately quantifying maternal distress levels specifically related to the disaster, which could be due to the confounding effect of multiple other factors, including unrelated personal experiences and previous mental health history.

With respect to the timing of the hurricane, our data indicates that children whose mothers were exposed during the second half of their pregnancy had higher methylation values, in the promoter region of the *LLRC39* gene, which is involved in the development of the sarcomeric M-band and may be associated with cardiac function. A recent study on adults who had been prenatally exposed to the Tangshan earthquake in 1976 also reported that those who had been exposed during the second trimester of pregnancy had significantly higher methylation levels in the promoter region of the human glucocorticoid gene NR3C1, and this finding was associated with poorer working memory performance^37^. Virk et al.^38^ found that the second trimester of pregnancy (13–27 weeks) was the most developmentally sensitive period for maternal bereavement, which acts through a similar pathway to stress, and this exposure was associated with higher risk for the development of type-2 diabetes later in life. Increased maternal cortisol levels during the second trimester of pregnancy have also been reported to be associated with decreased infant physical and neuromuscular maturation in males^39^.

HELiOS has collected extensive information on phenotypic characteristics of the children and potential confounders but we did not use these variables in the methylation analysis because of the small sample size. This may have biased our results, however, none of the mothers in the sample smoked either cigarettes or marijuana during pregnancy or heavily consumed alcohol. The small sample size is a limitation in this study, which decreased statistical power. To address this, we used the dmrff algorithm, which is more powerful than other comparable tools, to identify significant probes. The epigenetic findings of this study will guide our ongoing analysis evaluating the associations between the clinical outcomes and hurricane-related stressors within the entire HELiOS cohort, where we will include adjustments for confounders. Future studies are planned to evaluate the long-term clinical impact of hurricane Maria in the HELiOS cohort and to understand the underlying biological mechanisms that mediate the impact of early life stress on children’s health and development.

## Methods

### Study sample

Project HELiOS (Hurricane Exposures and Long-term Infant Outcomes Study) is a birth cohort that includes children who were prenatally exposed to Hurricane Maria in Puerto Rico or were conceived within three months post-disaster (born 09/20/2017 and 09/21/2018) and their mothers. The HELiOS cohort consists of 187 maternal-child dyads. For this study, the research team selected 16 children of mothers with symptoms of moderate to high depression and PTSD [high PNMS group]. These were matched by age and gender to 16 children of mothers with no symptoms of depression or PTSD (low PNMS group). The final sample for this study consisted of 32 children, 26 males, and 6 females, aged between 13 to 23 months at the time of the study (average 17.1 ± 4.3 months). There is a small number of females in the sample. Differences in autosomal DNA methylation by sex have been found. A list of significant probe IDs from this study was compared to the list of probe IDs associated with biological sex found by Grant et al.^40^. There was no overlap. The age of the mothers at the time of the study was between 22 and 41 years (average 31.2 ± 4.8 years). Children born to mothers with history of depression and PTSD were not excluded as we used the presence of the current symptoms as a proxy of how much stress the mother had during the pregnancy. When considering the effects of maternal mental health status, current evidence suggests that it is irrelevant if the symptoms are related to a first episode of Major Depression Disorder (MDD)/PTSD or if it is a recurrence as post-disaster, as literature shows both increased incidence and recurrence of both disorders. The study was conducted following the ethical principles for medical research involving human subjects as defined by the declaration of Helsinki. The study protocol is approved by the Institutional Review Board of the University of Puerto Rico Medical Sciences Campus (UPR-MSC, Protocol #A0060118). Written consent approved by the IRB of the UPR-MSC was obtained by the mothers or legal guardians.

### Study variables

Project HELiOS utilizes validated instruments to assess objective and subjective hurricane-related exposures, pregnancy outcomes, maternal and child medical histories, and child growth, development, and diet. In this report we included results for temporal, psychological, and selected hurricane exposure variables. Detailed pregnancy history, including pregnancy category and type of delivery was obtained using validated instruments currently used in other genetic cohort studies at the Dental and Craniofacial Genomics Core at the University of Puerto Rico. Gestational age (weeks) at the time of impact was used to assess the temporality of maternal exposure to the storm.

Maternal depressive symptoms were measured using the PHQ-9. The PHQ-9 is a multiple-choice, 4-point Likert scale, self-report inventory composed of nine items, assisting clinicians in screening for depression as well as selecting and monitoring treatment^41^. The PHQ-9 is a reliable and valid measure (α = 0.90) of depression severity^42^ and has been validated within primary care settings^42,43^. Its brevity makes it a useful clinical and research tool^42^ and it can detect and monitor depression in diverse ethnic/racial populations^44^. We have used scores of moderate depression or higher (score of 10 or higher) as indicator of depression. The Perceived Stress Scale (PSS-10) was used to assess subject maternal exposure to stress. The PSS-10 is widely used for measuring the degree in which situations in one’s life are perceived as stressful, unpredictable, and uncontrollable^45^. The PSS-10 was designed for at least a junior high school education. The questions are designed for any subpopulation group but have been previously used with pregnant women in post-hurricane settings^46^. The PSS-10 involves a 5-point Likert scale, with response ranging from 0 to 40, as higher scores indicate more stress. Scores from 0 to 13 are classified as low stress, 14–26 as moderate stress, and 27–40 as high stress. It has a good validity and reliability, with a α = 0.76^44^ and is used in Spanish in ongoing studies with Puerto Ricans. Post-traumatic stress disorder symptoms were measured using the PTSD checklist for DSM-5 (PCL-5), which is a 20-item self-report measure that assesses the 20 DSM-5 symptoms of PTSD 14. It takes approximately 5–10 min to complete. The total symptom severity score (range 0–80) is determined by summing the scores for each of the 20 items. We used the recommended cut-off for the PCL-5 of 33 points. We also used the criterion-based score (PCL-5 will be positive if the subject answers “moderately” or higher to all criteria needed for PTSD diagnosis). The PCL-5 has been translated into Spanish and has been used with Puerto Rican samples.

Measuring maternal mental health 1–2 years after Hurricane Maria is an ideal marker of PNMS as the moment of peak post-traumatic symptomatology is at this first-year mark^47^. Most cases of post-traumatic depression and PTSD improve over time, so the mothers that continue to present symptoms between 1 to 2 years after the hurricane are the more severe cases that will probably continue to present symptoms. Administration of certified psychological tests was performed by trained staff supervised by a licensed psychologist/psychiatrist from the Center for the Treatment and Management of Anxiety (CETMA) of the University of Puerto Rico Medical Sciences Campus. Any mothers who exhibited symptoms requiring special mental health evaluation and treatment were referred to CETMA.

To assess objective prenatal exposures related to Hurricane Maria we adapted the Exposure to Disaster Scale^48^ that was used with Hispanic populations after Hurricane Andrew in Florida and Hurricane Georges^49^ in PR. This self-report questionnaire includes questions assessing the threat to life or danger encountered during the hurricane, any injury or illness to self or others and the degree of property damage or loss. The adaptation includes specific stressors for Hurricanes Irma and María captured through formal interviews with patients receiving treatment for PTSD at the CETMA. These adaptations include measuring how long the person lived without electricity and communication services and the effect of the hurricane on sleep patterns.

Blood samples were collected by trained nurses/phlebotomists at the Hispanic Alliance for Clinical and Translational Research (Alliance) of the UPR Medical Sciences Campus. Samples were immediately transported to the Alliance core laboratory facility for processing and storage by trained staff. Whole blood samples were aliquoted, snap-frozen in dry ice/ethanol and stored at -80 0C. Dried blood spot (DBS) samples were also prepared in filtered paper for future analysis of environmental exposures, in addition to metabolic and cortisol tests.

### DNA methylation analysis

Coded blood samples were mailed to the Vieira Lab at the University of Pittsburgh for DNA methylation analysis. Samples were processed by the University of Pittsburgh Genomics Core. Genomic DNA (gDNA) was extracted from blood samples using the Qiagen DNeasy blood Mini Kit. Bisulfite modification of 1 µg DNA was conducted using an EZ DNA Methylation Kit (Zymo Research) according to the manufacturer’s procedure. The Infinium Methylation EPIC assay was performed according to Illumina’s standard protocol. Six sample pairs were processed on the same chip to account for chip-to-chip variation. All samples were run in duplicates. Infinium Methylation data was processed in R (version 4.0.2). Methylation levels of CpG sites were be calculated as β-values (β = intensity of the methylated allele (M)/ (intensity of the unmethylated allele (U) + intensity of the methylated allele (M) + 100)^50^. Data was preprocessed and analyzed according to the “A cross-package Bioconductor workflow for analyzing methylation array data”. All samples passed quality control (QC) with detection p-values > 0.05 and CpG coverage > 95% (Supplementary Fig. S5).

CpG sites (N = 11,648) on the sex chromosomes were not removed for the initial analysis. Data was normalized via the preprocess Funnorm function which performs better than other normalization methods on datasets with global methylation variation^51^. This function adjusts for known covariates via internal control probes, applies a background correction and corrects dye bias (Supplementary Fig. S6).

Probes with SNPs known to effect methylation at the probe site, or the single nucleotide extension were excluded. Cross-reactive probes were removed^52^. A cell heterogeneity correction was applied to normalized data^53^. To evaluate potentially confounding variables, MDS plots were created. Ethnicity and race affect methylation profiles^54,55^. In this dataset subjects did not cluster by race or ethnicity (Supplementary Fig. S7). The outliers in the MDS plots are samples with higher detection p-values.

Mode of delivery (Vaginal, C-section) may influence newborn methylation profiles, but the evidence is not conclusive^56^. In this dataset there is some clustering based on mode of delivery for subjects who delivered via un-scheduled C-section (Supplementary Fig. S8). This was used as a covariate in linear models to evaluate differential methylation. Preterm birth is also associated with differential methylation program in children^57^. In this dataset there was no clustering based on whether a delivery was term or preterm. A linear model was created via limma to assess the differential methylation by variable. All analyses were run using M-values, which have better statistical properties than beta values^58^. All tests were run using the false discovery rate (FDR) as a significance threshold^59^. To evaluate differential methylation the dmrff algorithm was used^60^. Like other popular methods of identifying differentially methylated sites, (bumphunter, comb-p, and DMRcate), dmrff uses EWAS summary statistics. Dmrff was chosen for this study because it combines these summary statistics from probes located in close proximity to each other. This takes into account that methylation marks at these sites is often interdependent, leading to is more power and better control of false positives than other tools. Data for DNase I hypersensitive sites (DHSs) is included for each significant site for which this data is available. DHSs indicate chromatin is not condensed at this location and is transcriptionally available.

## Supplementary Information

Supplementary Information.

## Data Availability

The datasets used and/or analyzed during the current study are available from the corresponding author on reasonable request.
