# Supplementary material for: Pre- and peri-natal hurricane exposure alters DNA methylation patterns in children
Source: Sci Rep. 2023 Mar 8;13:3875. doi: 10.1038/s41598-023-30645-5 (PMC9995354; doi:10.1038/s41598-023-30645-5)
Supplement: Supplementary file 1 — Supplementary Information. [file 41598_2023_30645_MOESM1_ESM.pdf]

Supplementary Materials

Title: Pre- and peri-natal hurricane exposure alters DNA methylation patterns in children

Authors: Erin Kello[1]†, Alexandre R. Vieira[1]†, Sona Rivas-Tumanyan[2], Maribel Campos-Rivera[2], Karen G. Martinez-Gonzalez[2‡], Carmen J. Buxó[2‡], Evangelia Morou-Bermúdez [2*‡]

Affiliations:

[1]University of Pittsburgh, Pittsburgh, USA

[2]University of Puerto Rico Medical Sciences Campus, San Juan, Puerto Rico

*Corresponding author. Email: evangelia.morou@upr.edu

†These authors contributed equally to this work

‡These authors contributed equally to this work

This PDF file includes:

Supplemental Table 1

Supplemental Figs. S1 to S8

Chromosome #	Adjusted p-value	ProbeID	Assocated Variable
1	0.046249494	cg26872588	PNMS
1	0.046249494	cg15324917	PNMS
10	0.006525317	cg05279086	PNMS
14	0.006043768	cg08182193	PNMS
3	0.006882115	cg16989784	PTSD (yes/no)
10	0.0047026	cg05279086	PTSD (yes/no)
10	0.048871044	cg26212957	PTSD (yes/no)
9	0.004214734	cg23727518	Total PHQ , categorical
9	0.006114372	cg26804081	Total PHQ , categorical
14	0.001043277	cg08182193	Total PHQ , categorical
16	0.013815236	cg19712499	Total PHQ , categorical
17	0.009862924	cg02238950	Total PHQ , categorical
1	0.042049482	cg26838150	Total PSS score, categorical
1	0.017017733	cg26295681	Total PSS score, categorical
6	0.021913176	cg19727175	Total PSS score, categorical
9	0.003805376	cg26089861	Total PSS score, categorical
12	0.042480243	cg15289144	Total PSS score, categorical
1	0.005211791	cg16829732	Gestational age in weeks on the day of hurricane
1	0.003632701	cg18118503	Gestational age in weeks on the day of hurricane
1	0.000492672	cg26117431	Gestational age in weeks on the day of hurricane
1	0.000492672	cg12187169	Gestational age in weeks on the day of hurricane
1	0.000492672	cg25754465	Gestational age in weeks on the day of hurricane
1	0.000492672	cg21821887	Gestational age in weeks on the day of hurricane
1	0.002309302	cg11819621	Gestational age in weeks on the day of hurricane
12	0.005842253	cg20527049	Gestational age in weeks on the day of hurricane
12	0.017561291	cg17314520	Gestational age in weeks on the day of hurricane
14	0.025564923	cg10257671	Gestational age in weeks on the day of hurricane
15	0.015077976	cg01096911	Gestational age in weeks on the day of hurricane
16	0.009202219	cg16470259	Gestational age in weeks on the day of hurricane
17	0.017946672	cg06360796	Gestational age in weeks on the day of hurricane
18	0.037704169	cg17550693	Gestational age in weeks on the day of hurricane
2	0.000434922	cg05964953	Gestational age in weeks on the day of hurricane
2	0.001683356	cg09330016	Gestational age in weeks on the day of hurricane
2	0.012861248	cg10477905	Gestational age in weeks on the day of hurricane
2	3.19463E-05	cg03756139	Gestational age in weeks on the day of hurricane
20	0.033190677	cg06815737	Gestational age in weeks on the day of hurricane
3	0.005112541	cg26296470	Gestational age in weeks on the day of hurricane
3	0.020803831	cg09929707	Gestational age in weeks on the day of hurricane
3	0.022048001	cg13062105	Gestational age in weeks on the day of hurricane
4	0.007475603	cg03539481	Gestational age in weeks on the day of hurricane
4	0.002829573	cg17472483	Gestational age in weeks on the day of hurricane

6	0.008502083	cg12674490	Gestational age in weeks on the day of hurricane
6	0.01118811	cg14815329	Gestational age in weeks on the day of hurricane
6	0.018835431	cg27270451	Gestational age in weeks on the day of hurricane
6	0.007962797	cg21078322	Gestational age in weeks on the day of hurricane
9	9.70465E-05	cg14041232	Gestational age in weeks on the day of hurricane
9	3.09616E-05	cg09187598	Gestational age in weeks on the day of hurricane

Supplemental Table 1: Significant differentially methylated single probes (DMPs) associated with all hurricane-related variables tested.

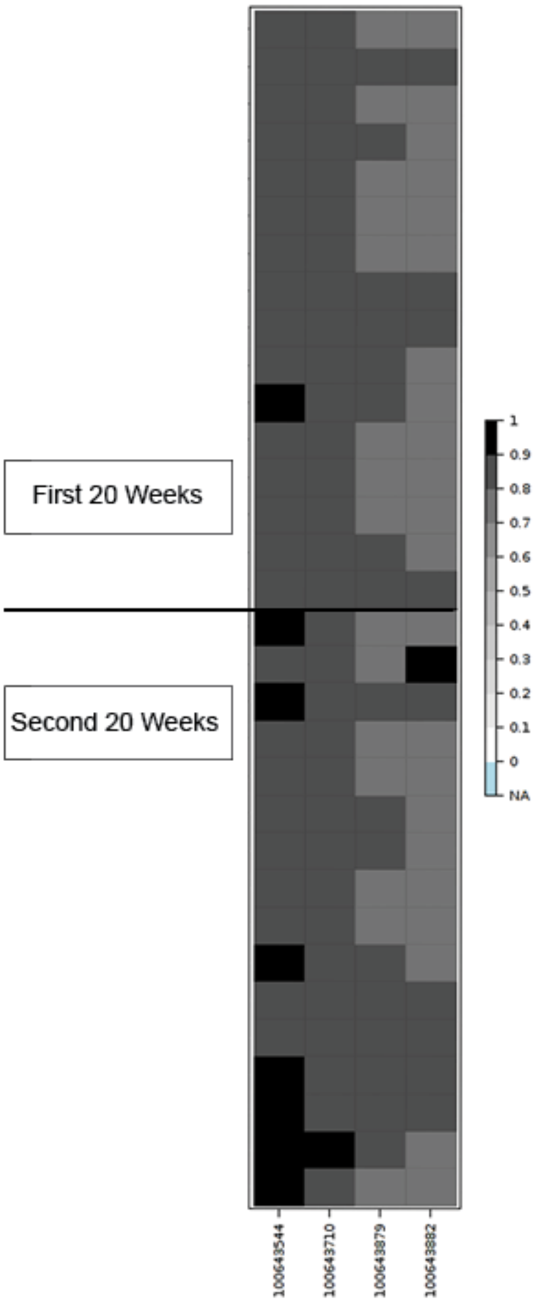

Supplemental Fig.S1 CoMET Heatmap of the four probes associated with the *LLRC39* DMR. Y-axis = by sample with designation of gestational time at the time of Hurricane Maria, 1=1st 20 weeks, 2= 2nd 20 weeks. X-axis = Probe ID. Darker colors indicate higher methylation levels.

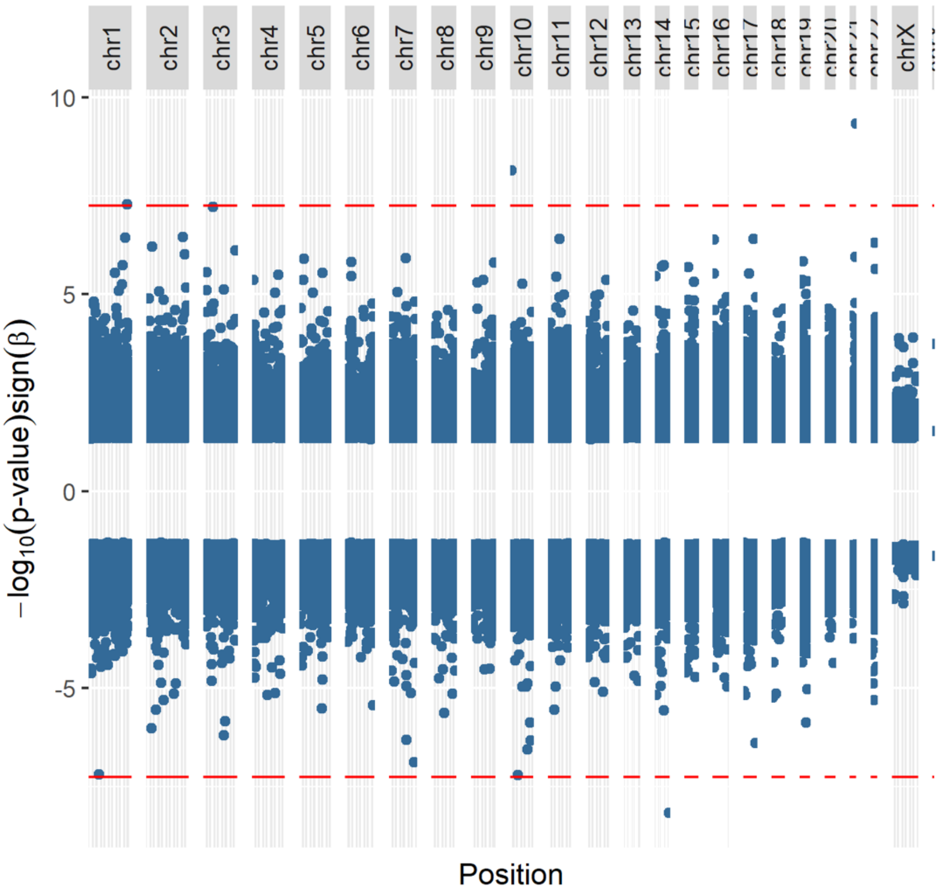

Supplemental Fig. S2 Manhattan plot of significant CpG sites associated with PNMS (yes/no). Red lines represent FDR significance threshold.

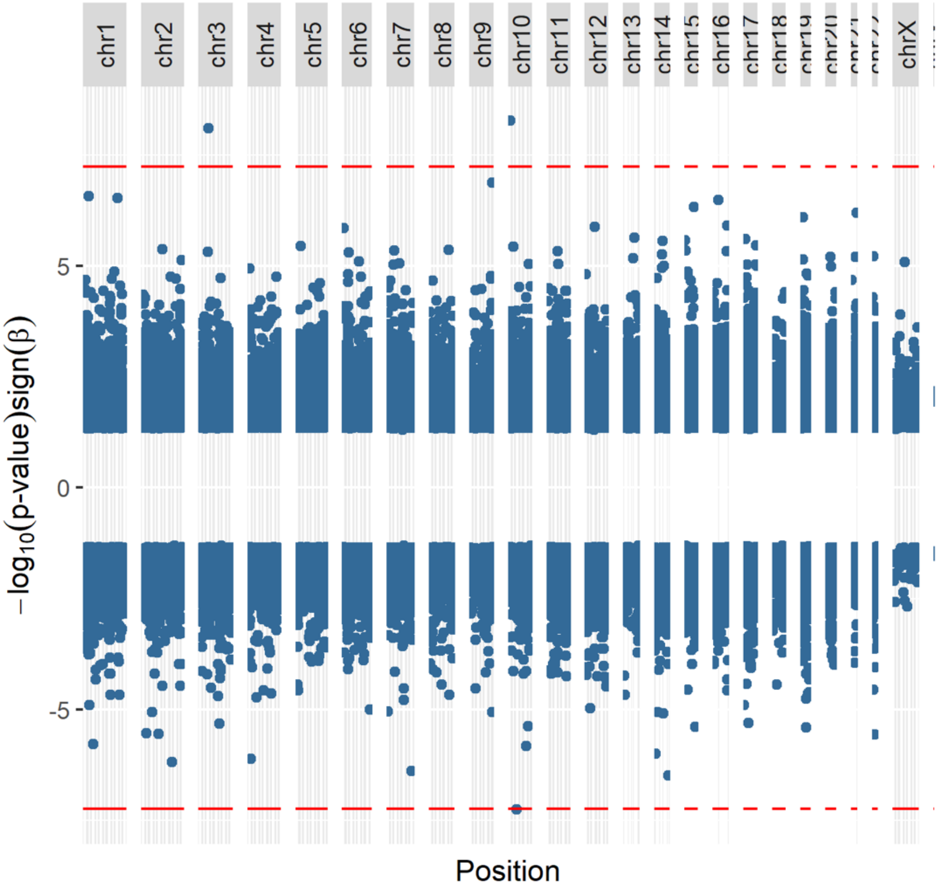

Supplemental Fig. S3 Manhattan plot of significant CpG sites associated with PTSD symptoms (yes/no). Red lines represent FDR significance threshold.

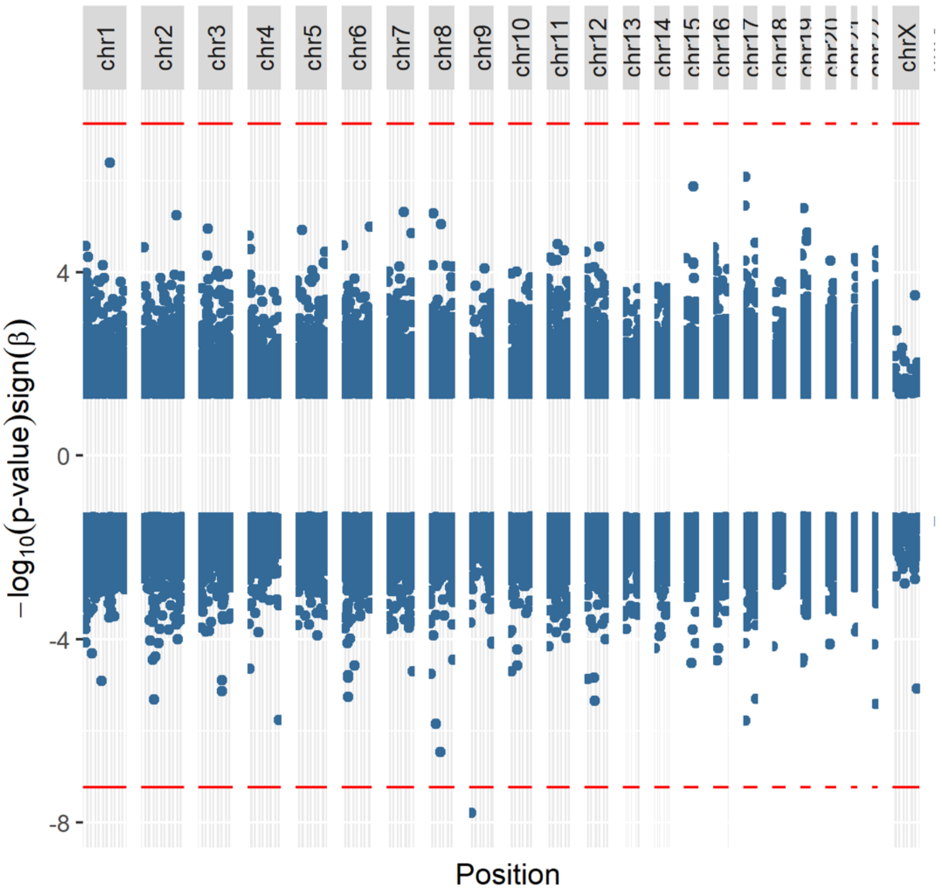

Supplemental Fig. S4 Manhattan plot of significant CpG sites associated with property damage score. Red lines represent FDR significance threshold.

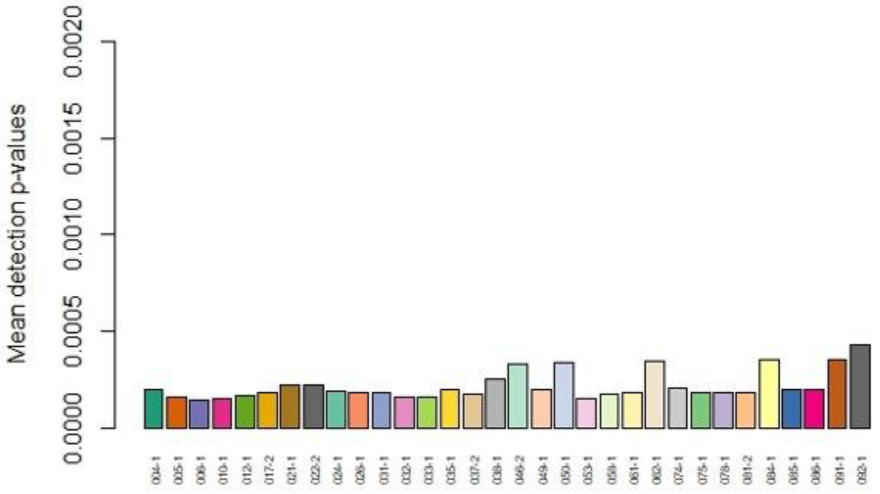

Supplemental Fig. S5 Mean detection p-values for all samples by number.

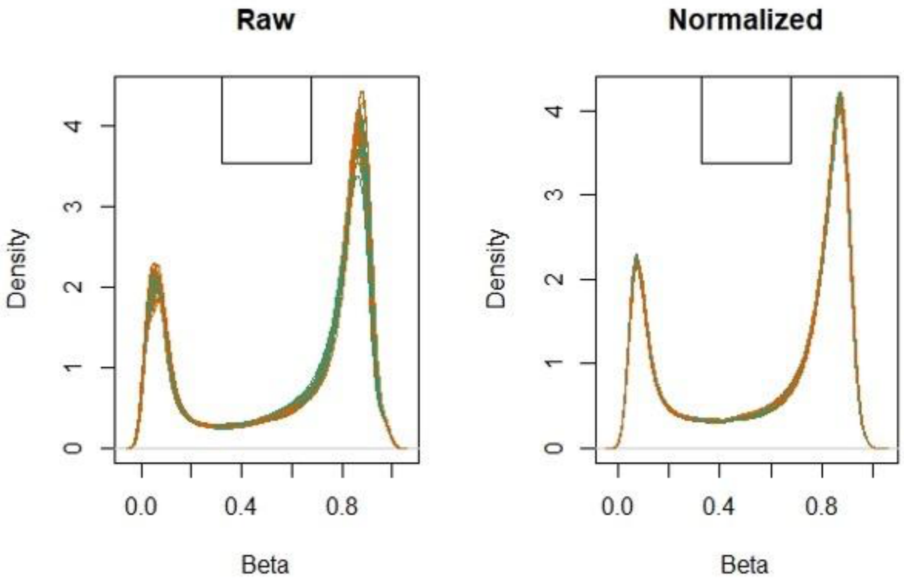

Supplemental Fig. S6. Density plots before and after normalization.

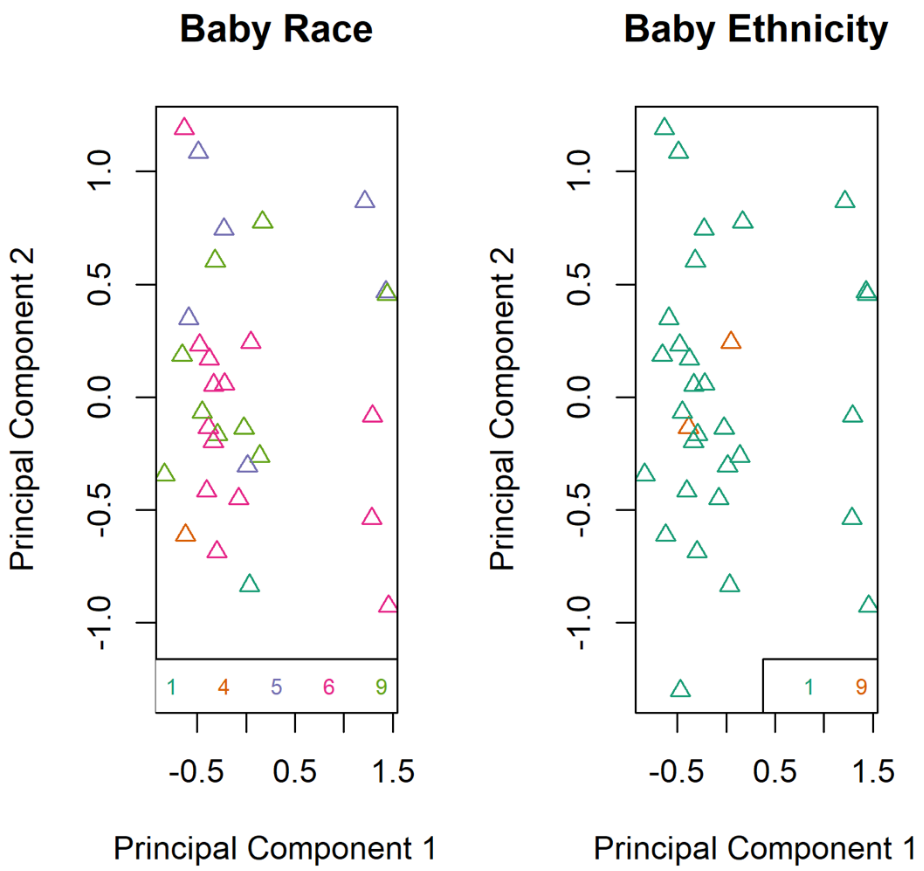

Supplemental Fig. S7 MDS plots of subjects by race and ethnicity. **Race plot**: 1: American Indian / Alaska Native; 4: Black/African American; 5: White/Caucasian; 6: More than 1 race; 9: Don't know/no response. **Ethnicity plot**: 1: Hispanic or Latino; 9: Don't know / No response.

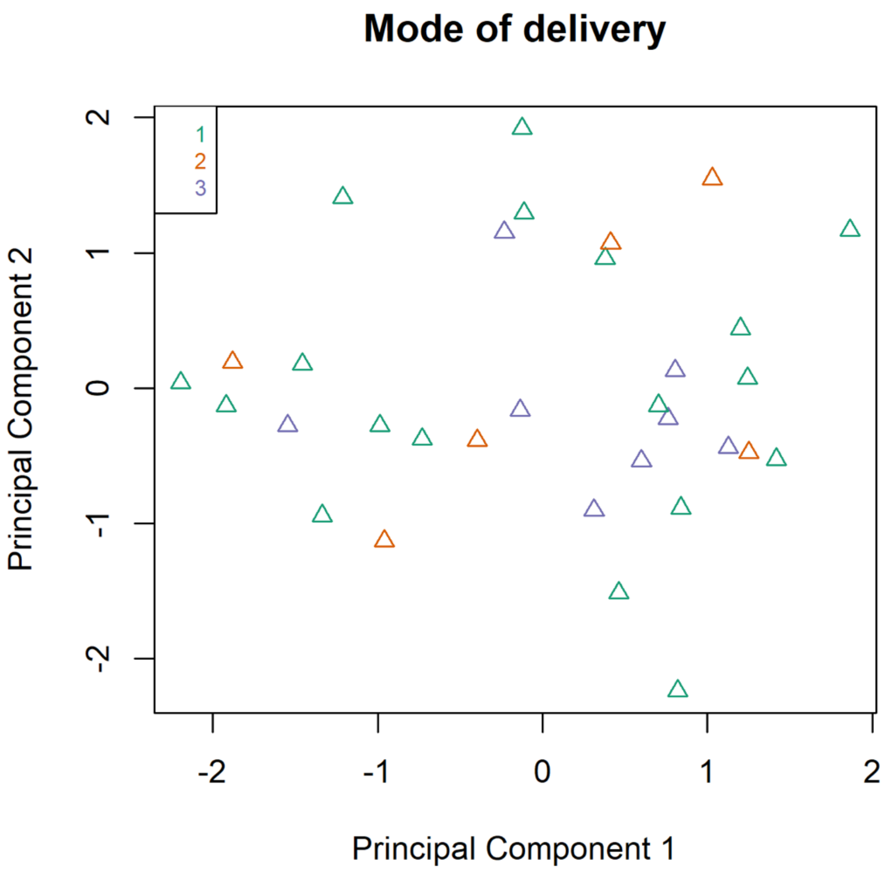

Supplemental Fig. S8. MDS plot of mode of delivery: 1: Vaginal delivery; 2: C-section, scheduled; 3: C-section, unscheduled.
